# Intron 4–5 *hTERT* DNA Hypermethylation in Merkel Cell Carcinoma: Frequency, Association with Other Clinico-pathological Features and Prognostic Relevance

**DOI:** 10.1007/s12022-021-09669-y

**Published:** 2021-04-28

**Authors:** Costantino Ricci, Luca Morandi, Francesca Ambrosi, Alberto Righi, Dino Gibertoni, Francesca Maletta, Claudio Agostinelli, Angelo Gianluca Corradini, Silvia Uccella, Silvia Asioli, Fausto Sessa, Stefano La Rosa, Mauro Giulio Papotti, Sofia Asioli

**Affiliations:** 1 Pathology Unit, Maggiore Hospital, AUSL Bologna, Bologna, Italy; 2 Department of Experimental, Diagnostic and Specialty Medicine (DIMES), University of Bologna, Bologna, Italy; 3 Department of Biomedical and Neuromotor Sciences, Functional MR Unit, IRCCS Istituto delle Scienze Neurologiche, Bologna, Italy; 4 Department of Pathology, IRCCS Istituto Ortopedico Rizzoli, Bologna, Italy; 5 Department of Biomedical and Neuromotor Sciences, Unit of Hygiene and Biostatistics, University of Bologna, Bologna, Italy; 6 Department of Oncology, University of Turin, Città Della Salute Hospital, Turin, Italy; 7 Unit of Pathology, Department of Medicine and Surgery, University of Insubria, Varese, Italy; 8 Unit of Pathology, Morgagni-Pierantoni Hospital, Forlì, 47121 Italy; 9 Institute of Pathology, Lausanne University Hospital and University of Lausanne, Lausanne, Switzerland; 10 Haematopathology Unit, IRCCS Azienda Ospedaliero-Universitaria di Bologna, Bologna, Italy; 11 Department of Biomedical and Neuromotor Sciences (DIBINEM) Surgical Pathology Section- Alma Mater Studiorum , University of Bologna , Bologna, Italy

**Keywords:** Merkel cell carcinoma, Merkel cell polyomavirus, Methylation, *HTERT*, *HTERT intron 4–5*, Rs10069690, Telomerase

## Abstract

Merkel cell carcinoma (MCC) is an aggressive skin tumor with neuroendocrine differentiation, mainly affecting elderly population or immunocompromised individuals. As methylation of the human telomerase reverse transcriptase (m*hTERT*) has been shown to be a prognostic factor in different tumors, we investigated its role in MCC, in particular in intron 4–5 where rs10069690 has been mapped and recognized as a cancer susceptibility locus. DNA methylation analysis of *hTERT* gene was assessed retrospectively in a cohort of 69 MCC patients from the University of Bologna, University of Turin and University of Insubria. Overall mortality was evaluated with Kaplan-Meier curves and multivariable Royston-Parmar models. High levels of m*hTERT* (m*hTERT*_high_) (HR = 2.500, *p* = *0.015*) and p63 (HR = 2.659, *p* = *0.016*) were the only two clinico-pathological features significantly associated with a higher overall mortality at the multivariate analysis. We did not find different levels of m*hTERT* between MCPyV (+) and (−) cases (21 vs 14, *p* = *0.554*); furthermore, m*hTERT*_high_ was strongly associated with older age (80.5 vs 72 years, *p* = *0.026*), no angioinvasion (40.7% vs 71.0%, *p* = *0.015*), lower Ki67 (50 vs 70%, *p* = *0.005*), and PD-L1 expressions in both tumor (0 vs 3%, *p* = *0.021*) and immune cells (0 vs 10%, *p* = *0.002*). m*hTERT* is a frequently involved epigenetic mechanism and a relevant prognostic factor in MCC. In addition, it belongs to the shared oncogenic pathways of MCC (MCPyV and UV-radiations) and it could be crucial, together with other epigenetic and genetic mechanisms as gene amplification, in determining the final levels of *hTERT* mRNA and telomerase activity in these patients.

## Introduction

Merkel cell carcinoma (MCC) is an aggressive neuroendocrine tumor, predominantly affecting elderly population or immunocompromised individuals [1, 2]. Although MCC is rare, its annual incidence has significantly increased in the last decades, probably due to population aging and the increase of risk factors as chronic sun damage and immunosuppressive therapies [3, 4]. Despite aggressive therapeutic strategies, MCC has a 5-year overall mortality of about 33%, more than twice than that of cutaneous melanoma [4]. Therefore, deep insight into its genetic background could be crucial to improve targeted therapeutic strategies and outcomes of MCC patients. In the last years, several authors investigated the most commonly altered genes and genetic pathways (*TP53*, *RB1*, *PIK3CA*, *KIT*, *PDGFRA*, *PDCD1*, chromosomal abnormalities, miRNAs, and many others) in MCC [5–12]. They found that a number of oncogenes and tumor-suppressor genes, even if not mutated, are abnormally up- or down-regulated in MCC, through different epigenetic mechanisms [5–12]. Telomerase (TERT), an RNA-dependent DNA polymerase, represents one of the crucial steps in the malignant transformation of several tumors, stabilizing the telomere length and immortalizing cancer cells [9, 13–15]. The functional catalytic subunit of TERT (human telomerase reverse transcriptase-hTERT), is up-regulated through multiple genetic and epigenetic mechanisms, including *hTERT* promoter mutations, amplifications, promoter methylation, alternative splicing, and many others [9, 13–15]. Although *hTERT* has a well-established pathogenetic and prognostic role in several tumors, it has been poorly investigated in MCC and little is known about its function in this neoplasia [9, 16, 17]. Xie H. et al. showed that there is a widespread *hTERT* mRNA expression in MCC and that higher *hTERT* mRNA levels are associated with a significantly shorter overall survival [9]. Nevertheless, they only investigated *hTERT* promoter mutations and amplifications and, as clarified by the same authors, the complex genetic and epigenetic regulation system of *hTERT* expression in MCC is still largely unknown [9]. Genome-wide association studies (GWAS) have recently mapped risk alleles for at least 10 cancer types in a region of chromosome 5 (5p15.33), harboring *hTERT* gene [18]. Allele-specific effects on DNA methylation were seen in this region identifying the single-nucleotide polymorphism (SNP) biomarker rs10069690 as a possible effector on the methylation and subsequently on gene expression [18, 19]. In this work, we decided to evaluate epigenetic modifications with regard to DNA methylation in this precise region not located on the promoter but within the intron (m*hTERT*) [18, 19]. We previously characterized DNA methylation alterations in this locus in a large cohort of oral squamous cell carcinoma, identifying the most correlated CpG located at Chr5:1279643-1279644, with an area under the curve (AUC) of 0.92 [18, 19]. Since m*hTERT* could be a relevant epigenetic mechanism in *hTERT* expression and an independent prognostic factor in several tumors, we investigated whether m*hTERT* is associated with overall mortality and with the most relevant clinical-pathological features in a series of MCC patients [9, 13–32].

## Materials and Methods

### Ethics Statement

All clinical investigations were conducted according to the principles of the Declaration of Helsinki. The study was approved by the Institutional Review Board and the local ethics committee (study number CE18083, DIBINEM-UNIBO-rif.CE AVEC number 377/2018/OSS/AUSLBO). All information regarding the human material used in this study was managed using anonymous numerical codes.

### Patients and Specimens

This is a multicentric study enrolling 69 subjects from three different Italian Universities: University of Turin (31 cases), University of Bologna (31 cases), and University of Insubria (7 cases). This cohort has been previously published by our group in a study on m*PDCD1*, and the follow-up has been updated [8]. All tumors were re-staged according to the 8^th^ edition of the AJCC Cancer Staging Manual [4]. Tissues were fixed in 10% formalin, embedded in paraffin, and stained with hematoxylin and eosin (H&E). Slides from each case were reviewed by five pathologists (S.A., S.U., S.L.R., F.M., C.R.) to confirm the diagnosis and restage the tumors, to assess pathological parameters, and to choose one representative paraffin block for additional analyses. We evaluated the immune cells (ICs) rather than the tumor-infiltrating lymphocytes (TILs), as previously reported [33–35].

### Immunohistochemistry

Immunohistochemistry (IHC) was performed on archival, formalin-fixed, paraffin-embedded tissues, using the following antibodies: PD-L1 (clone 123 C3SP142, Ventana-Diapath, dilution 1:50), CD3 (clone 2GV6, Ventana-Diapath, dilution RTU), Cytokeratin 20 (clone SP33, Ventana, dilution RTU), Chromogranin (clone LK2H10, Ventana, dilution RTU), TTF-1 (clone 847G3/1, Ventana, dilution RTU), and Ki67 (clone MIB-1, Dako, dilution 1:100). PD-L1 positivity was assessed on ICs and tumor cells (TCs), as previously described by Lipson et al. [34].

### DNA Mutation Analysis of *hTERT* Promoter

#### DNA Purification

DNA from macrodissected tumor tissue was digested at 56 °C for 3 h to over-night using the Quick Extract™ FFPE DNA extraction kit (Epicentre, Madison, WI, USA). A denaturation step at 95 °C for 5 min, followed by centrifugation at 10,000×*g* at 4 °C for 5 min, was adopted to pellet the undigested tissue and solidify the paraffin afloat. DNA was collected from the interphase and stored at 4 °C. *hTERT* promoter mutation analysis was performed by next-generation sequencing (NGS) using a two-step PCR protocol, as previously described [36]. The region of interest spanned the two most common mutations including C228T (c.-124C>T) and C250T (c.-146C>T) (Chr5: 1295035-1295199 keeping hg38 as a reference genome version). Each NGS experiment was designed to allocate at least 2 k reads/region aimed to have a depth of coverage of at least 2000×. Reads were mapped in a Galaxy Project environment to the hg38 human reference genome with Bowtie2, GATK local realignment, HaplotypeCaller, and Picard MarkDuplicates [37]. Mutations were visualized using BAM files loaded onto the Integrative Genomic Viewer (IGV); only mutations with a variant allele frequency (VAF) threshold > 5% were reported.

### Quantitative DNA Methylation Analysis of *hTERT* (m*hTERT*)

#### Bisulfite DNA Treatment

Bisulfite treatment of genomic DNA was carried out with the EZDNA Methylation-Lightning™ Kit (Zymo Research, cod. D5031) according to the manufacturer’s protocol.

#### Bisulfite Next-Generation Sequencing analysis

Quantitative DNA methylation analysis was performed by bisulfite-next-generation sequencing as previously described [19]. The library was prepared in two steps: a first multiplex PCR amplification for target enrichment and a second round of amplification for barcoding which was performed using the Nextera™ index kit. MethPrimer (http://www.urogene.org/cgi-in/methprimer/methprimer.cgi) was applied to identify CpGs and the best primers of choice for *hTERT* [38]. Three regions of interest were evaluated: the promoter region A as indicated previously by Svahn et al. with the following mapping coordinates: Chr5: 1295506-1295709, the promoter region B with mapping coordinates Chr5: 1295027-1295220, and an additional region previously described by Morandi et al. spanning from Chr5: 1.279.604 to 1.279.759 (intron-4–5) (keeping hg38 as a reference) (*mhTERT*) [19, 39]. Six different CpGs are located in this region: 1279732, 1279723, 1279714, 1279660, 1279643, 1279631. Amplicons were purified using MagSi-NGSPREP beads (Magnamedics, Geleen, The Netherlands), quantified with Fluorometer Quantus™ (Promega, Madison, WI, USA), and then pooled and loaded on Illumina MiSEQ (Illumina, San Diego, CA, USA) according to the manufacturer’s protocol.

#### Bisulfite Sequencing Data Analysis

Each NGS experiment was designed to allocate at least 2 k reads/region, in order to have a depth of coverage of about 2000× to allow a good estimate of DNA methylation level. The methylation ratio of each CpG was calculated in a Galaxy Project environment (Europe) using BWA-meth for mapping and generating the bam file followed by the MethylDackel tool [37]. In parallel, DNA methylation level and quality control of various FASTQ files were evaluated using the web-tool EPIC-TABSAT [40].

#### Statistical Analysis

**Fig. 1:**
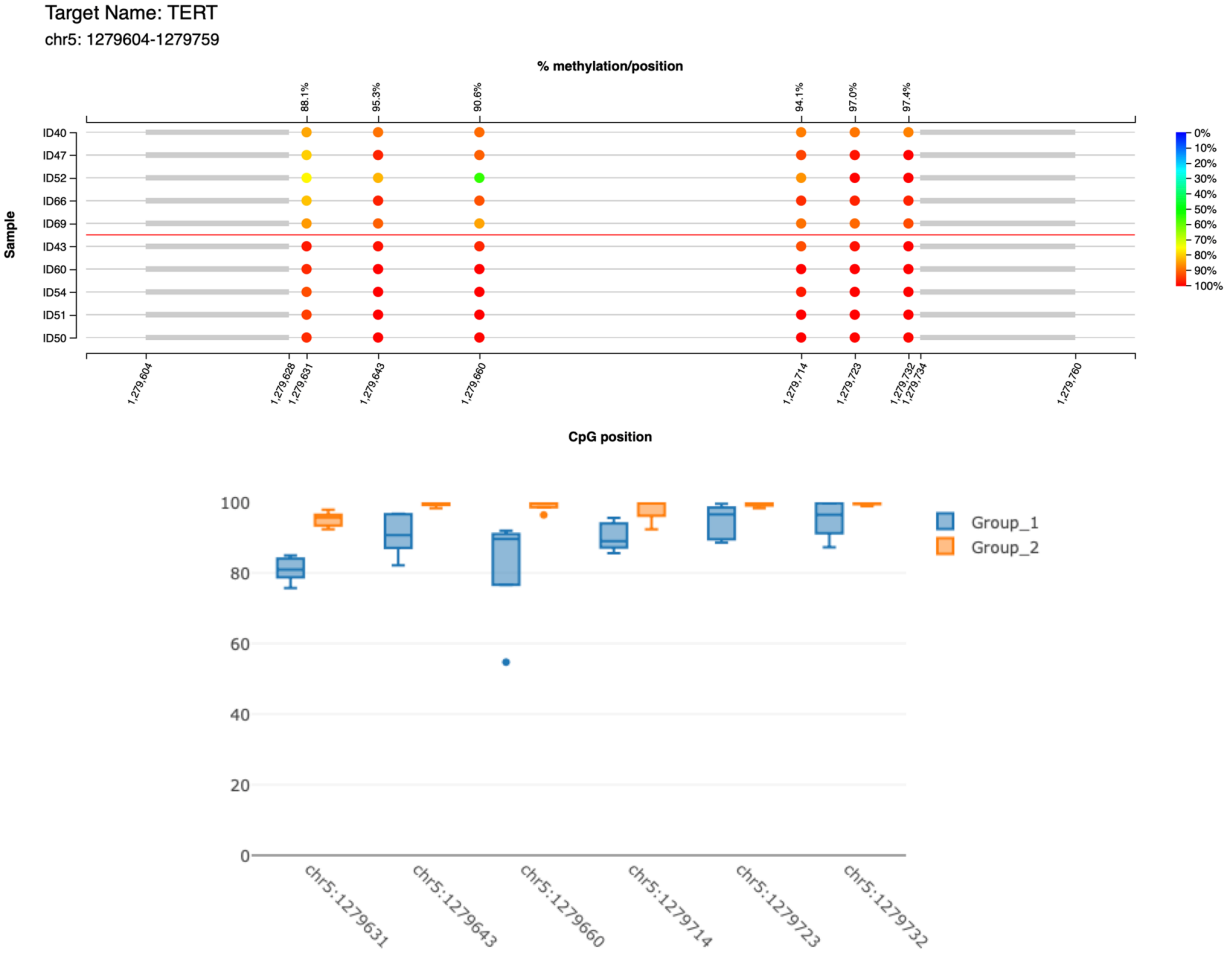
The lollipop graph displays the percent methylation per CpG position for 10 randomly selected samples, 5 m*hTERT*_high_ cases, and 5 m*hTERT*_low_ ones, respectively. The color of the circles represents the percent methylation as shown in the color legend on the right side of the plot. On the lower *x*-axis, the genomic positions of the CpGs are displayed, the upper *x*-axis shows the summarized average percent methylation for this CpG position

*hTERT* methylation levels (m*hTERT*) have been dichotomized according to the obtained median value (0.97) chosen as threshold (Fig. 1). In brief, the tumor was stratified in m*hTERT*_high_ if the methylation levels of the CpGs mapped on Chr5:1279643-1279642, Chr5:1279660-1279659, Chr5:1279714-1279713, Chr5:1279723-1279722 were all above the cut-off threshold (0.97); on the contrary, m*hTERT*_low_ was assigned if even just one was below the threshold (range of methylation = 0.33–1.000). Continuous variables were dichotomized according to sensible cutoffs in order to be used for survival analysis. Patients have been aggregated in two groups for age ≥ or < 75 years (corresponding to the population mean age), tumor size ≥ 2 cm, mitosis ≥ 10/high-power field (HPF), Ki67 ≥ 50%, tumor thickness ≥ 10 mm. Because Ki67 dichotomized according to cut-off of < 55%/≥ 55% was not an independent predictor of survival at the multivariate analysis as recently reported by our group, we adopted the cut-off of < 50%/≥ 50% according to our previous data [41–43]. Stage was dichotomized into two classes, I-II (low stages) and III-IV (high stages). Patients’ characteristics were compared across m*hTERT* status using chi-square or Mann-Whitney test. Overall survival (OS) was evaluated by means of incidence rate ratios and survival analysis using Kaplan-Meier curves and multivariable Royston-Parmar (RP) models. These models have the advantage, over the more traditional Cox regression approach, to allow a greater flexibility in the specification of the baseline log cumulative survival function, which provides a better fit to the data and, as a consequence, an improvement of model accuracy [44, 45]. RP models use restricted cubic splines with an appropriate number of knots to fit even complex mathematical functions that best represent the survival function inherent to the data [44, 45]. We selected the most appropriate functional form among proportional hazards models, proportional odds models, and probit models with 1 to 5 evenly spaced knots, based on the lowest values of the Akaike information criterion (AIC) and Bayesian information criterion (BIC) statistics, which denote the better fit to the data. After identifying the survival function, the multivariable fractional polynomial (MFP) procedure was used to select the variables influencing OS from a set of initial candidate predictors. MFP automatically performs backward selection by removing the weakly significant predictors and identifies the most appropriate functional form of the relationships between the outcome and the continuous covariates, thus improving model accuracy [46]. The initial model was estimated using m*hTERT* status as main exposure and age, gender, tumor size, tumor thickness, angioinvasion, p63, MCPyV, clinical stage III-IV, PD-L1 in TCs ≥ 5%, and infiltrative growth as covariates and obtaining robust standard errors for patients clustering into centers. In the backward selection process of MFP, m*hTERT* was imposed to be retained in the model, as well as covariates with *p* < 0.100. This confidence level was chosen in order to keep in the final model the variables that may not reach a *p* value lower than the standard 0.05 confidence level only because of the small sample size, although showing a remarkable effect on OS. Lastly, the model-adjusted survival curves of m*hTERT* and the retained clinical-pathological parameters were estimated. All analyses have been carried out using Stata v.15.1, specifically the stpm2 procedure was used to estimate RP models [47].

## Results

### Patients Characteristics

The clinico-pathological features of the cohort have already been described in a previous study [8]. In brief, the study cohort included a total of 69 patients, 38 (55.1%) males, and 31 (44.9%) females, with a median age of 77 years. According to the 8^th^ edition of the AJCC Cancer Staging Manual, localized disease (AJCC Clinical Stage I-II) was present in 45 (65.2%) and advanced (AJCC Clinical Stage III-IV) in 24 (34.8%) patients, with only two (2.9%) patients displaying distant metastases (AJCC Clinical Stage IV) [4]. Pathological stage was available only for 28 patients (41%) because only after 2010, the SLNB has been recommended in all patients with localized disease [4, 48]. Patients were followed up for a maximum time of 192 months. At the end of follow-up, 39 (56.5%) patients died, 35 (50.7%) due to MCC (DOD) and 4 (5.8%) of other causes (DOC). The median follow-up time was 24 months (range 2–150) for DOD, 17 months (range 14–60) for DOC, and 46.5 months (range 6–192) for patients still alive at follow-up. At the end of follow-up, 9 patients (13.0%) were alive with disease progression (AWD) and 21 ones (30.4%) had no evidence of disease (NED).

### hTERT Promoter Mutation Analysis

Only three cases showed the *hTERT* promoter mutation C250T (c.-146C>T) with a variant allele frequency below 7% for all.

### DNA Methylation of Intron 4–5 (m*hTERT*) and Correlation with Clinico-pathological Features

**Fig. 2:**
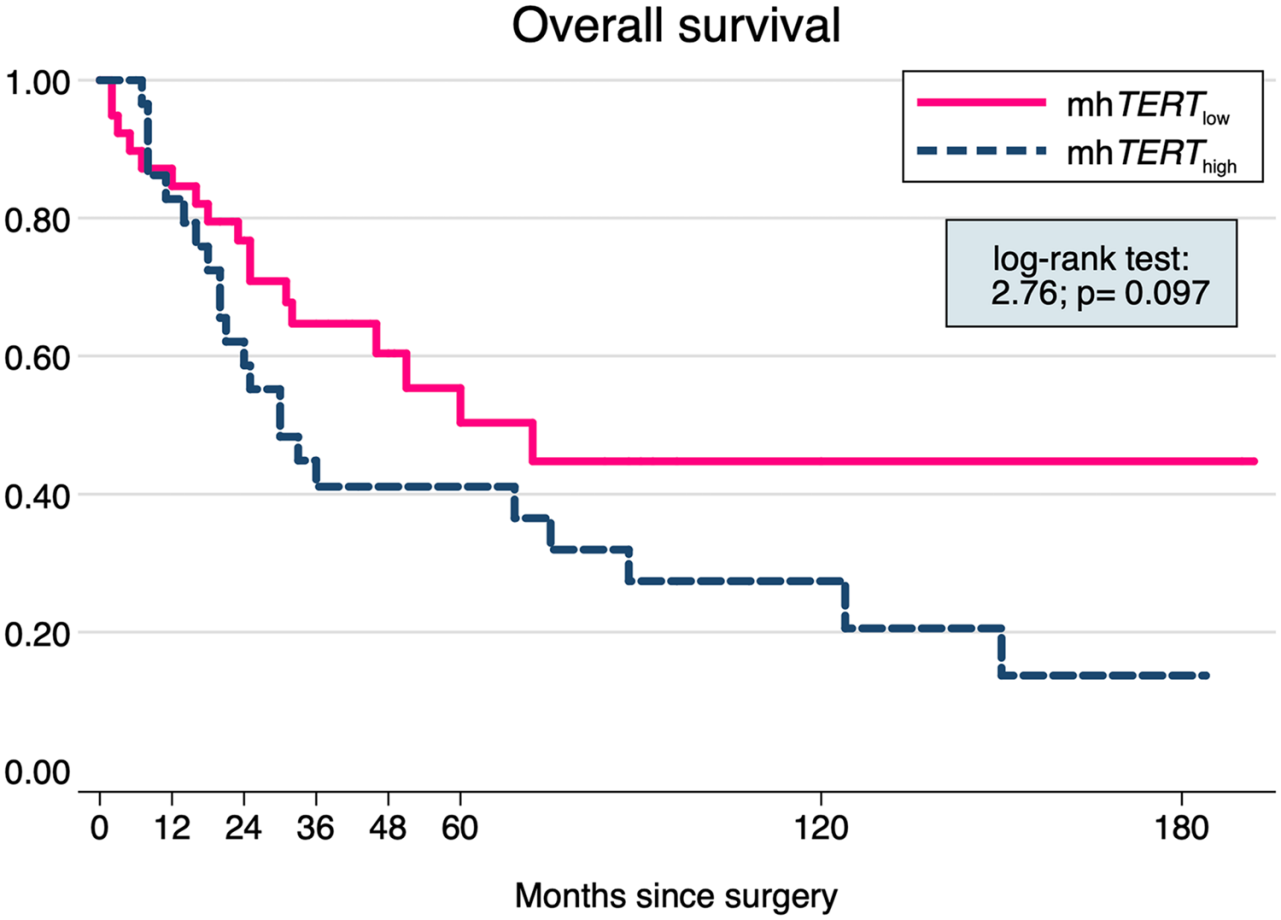
Kaplan-Meier survival curve shows a higher mortality in m*hTERT*_high_ group (log-rank test: *t* = 2.76, *p* = *0.097*), with the curves diverging about 12 months after the surgery

**Fig. 3:**
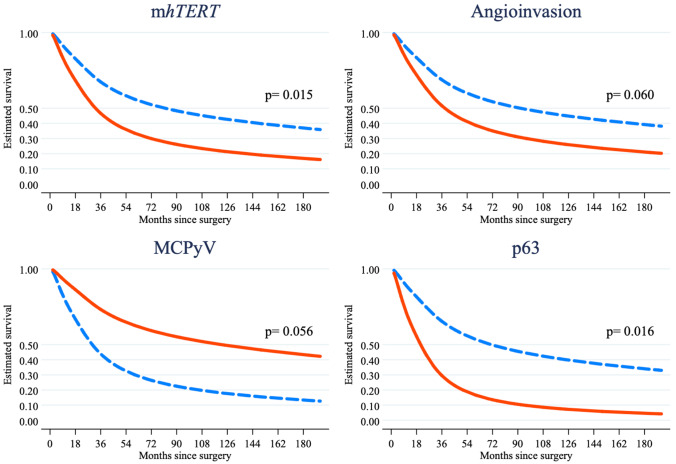
In the final multivariable RP model, mh*TERT*_high_ (*p* = *0.015*) and p63 (*p* = *0.016*) strongly influenced overall survival, whit borderline-significant associations for angioinvasion (*p* = *0.060*) and absence of MCPyV (*p* = *0.056*). Blue-dashed line: mh*TERT*_low_ or absence of examined parameter (p63, angioinvasion and MCPyV); red line: mh*TERT*_high_ or presence of examined parameter (p63, angioinvasion and MCPyV)

The study population was composed by 30 (43.5%) patients with m*hTERT*_high_ and 39 (56.5%) patients with m*hTERT*_low_, whose clinical-pathological features are summarized in Table 1. Patients with m*hTERT*_high_ differed significantly for an older median age (80.5 vs 72 years, *p* = *0.026*), but a lower angioinvasion (40.7% vs 71.0%, *p* = *0.015*), Ki67 expression (50 vs 70%, *p* = *0.005*) and PD-L1 expressions in both TCs (0 vs 3%, *p* = *0.021* as continuous variable; 7 vs 17, *p* = *0.080* with cut-off of 5%), and ICs (0 vs 10%, *p* = *0.002* as continuous variable; 7 vs 23, *p* = *0.006* with cut-off of 5%). There was no difference in the distribution of m*hTERT* levels between MCPyV (+) and (−) cases (21 vs 14, *p* = *0.554*). Among the 39 patients with a fatal outcome, 22 (56.4%) were in the m*hTERT*_high_ group and 17 (43.6%) in the m*hTERT*_low_ one. The mortality incidence ratio of m*hTERT*_high_/m*hTERT*_low_ was 1.648 (95% CI: 0.836–3.305), suggesting higher, although not statistically significant, mortality in m*hTERT*_high_ group. The Kaplan-Meier survival analysis confirmed this result (log-rank test: *t* = 2.76, *p* = *0.097*; Fig. 2) and showed that the curves diverge about 12 months after the surgery. Other clinical-pathological features associated to a higher mortality risk in the bivariate analysis were clinical stage III-IV (log-rank test: *t* = 4.16, *p* = *0.041*), p63 (log-rank test: *t* = 13.12, *p* < *0.001*), and absence of MCPyV (log-rank test: *t *= 11.62, *p* = *0.001*). In the final multivariable RP model, m*hTERT*_high_ was confirmed as associated to a lower OS (HR = 2.500, *p* = *0.015*) also after adjusting for p63 (HR = 2.659, *p* = *0.016*) absence of MCPyV (HR = 0.478, *p* = *0.056*) and angioinvasion (HR = 2.168, *p* = *0.060*), while all the other covariates were removed because they did not show a remarkable influence on OS (*p* > 0.100) (Table 2 and Fig. 3).

**Table 1:** Clinical-pathological data of the study population, divided in m*hTERT*_low_ and m*hTERT*_high_ groups

	*n*	Study population, *n* (% or range)	m*hTERT*_low_,* n* (% or range)	m*hTERT*_high_,* n* (% or range)	*χ*^2^ test; *p*
Study population	69	69(100.0)	39(56.5)	30(43.5)	
Age, median(range)	69	77(41–95)	72(41–95)	80.5(59–89)	− 2.22; 0.026^a^
Age ≥ 75 years	69	41(59.4)	17(43.6)	24(80.0)	9.32; 0.002
Males	69	38(55.1)	22(56.4)	16(53.3)	0.06; 0.799
Size, median(range)	65	2.5(0.3–8.5)	2.5(0.3–6.5)	2.4(0.6–8.5)	0.440; 0.660^a^
Size ≥ 2 cm	69	38(55.1)	23(59.0)	15(50.0)	0.55; 0.458
Clinical stage III-IV	69	24(34.8)	13(33.3)	11(36.7)	0.08; 0.773
Tumor thickness, mm	65	12(1–45)	12.5(1–22)	10(1–45)	1.13; 0.257^a^
Tumor thickness ≥ 10 mm	65	37(56.9)	24(63.2)	13(48.1)	1.45; 0.228
Angioinvasion	65	38(58.5)	27(71.0)	11(40.7)	5.97; 0.015
Mitosis ≥ 10/HPF	69	40(58.0)	21(53.8)	19(63.3)	0.63; 0.429
p63-IHC	69	13(18.8)	7(17.9)	6(20.0)	0.05; 0.829
MCPyV	69	35(50.7)	21(53.8)	14(46.7)	0.35; 0.554
Ki67, median(range)	69	60(10–100)	70(30–100)	50(10–95)	2.83; 0.005^a^
Ki67 ≥ 50%	69	42(60.9)	28(71.8)	14(46.7)	4.50; 0.034
ICs	65	34(52.3)	23(60.5)	11(40.7)	2.48; 0.116
Infiltrative growth	65	22(33.8)	16(42.1)	6(22.2)	2.79; 0.095
PD-L1 ICs	65	3(0–70)	10(0–70)	0(0–50)	3.11; 0.002^a^
PD-L1 TCs	69	1(0–30)	3(0–30)	0(0–20)	2.31; 0.021^a^
Site	69				1.36; 0.508
Head and neck		26(37.7)	14(35.9)	12(40.0)	
Extremities		28(40.6)	18(46.2)	10(33.3)	
Trunk		15(21.7)	7(17.9)	8(26.7)	

*n* number of patients for which clinical-pathological features were present and/or evaluated, *ICs* immune cells, *TCs* tumor cells, *HPF* high-power filed, *p63* protein p63, *IHC* immunohistochemistry, *MCPyV* Merkel cell polyomavirus, *Ki67* proliferation index Ki67/MIB1, *PD-L1* programmed death-ligand 1, *mhTERT*_*low*_ low level of m*hTERT*, *mhTERT*_*high*_ high level of m*hTERT*

^a^Mann-Whitney test

**Table 2:** Results of the multivariable Royston-Parmar model (*n* = 65)

	HR	95% CI	*p* value
m*hTERT*_*high*_	2.500	1.193–5.236	0.015
Angioinvasion	2.168	0.966–4.863	0.060
p63-IHC	2.659	1.203–5.877	0.016
MCPyV	0.478	0.224–1.020	0.056

*CI* confidence interval, *HR* hazard ratio, *mhTERT*_*high*_ high level of m*hTERT*,* p63* protein p63, *IHC* immunohistochemistry, *MCPyV* Merkel cell polyomavirus

### hTERT Promoter Methylation

DNA promoter methylation for regions A and B as previously described was available in only 13 cases [39]. This was due for overfixation problems, bisulfite treatment and for the amplicon length, which was higher than for intron 4–5 locus. Region A, spanning from Chr5: 1295506 to 1295709, revealed full methylation in all cases except for two, both showing m*hTERT*_low_. Region B spanning from Chr5: 1295027 to 1295220 was found to be hypomethylated (ranged from 50 to 0% methylation) in all cases. Due to lack of data for most of cases, additional statistical analysis was not performed.

## Discussion

Three main findings emerge from this study on m*hTERT* in MCC:

1. m*hTERT* is an epigenetic mechanism frequently activated in MCC, in particular in a locus mapped on intron 4–5 around the SNP variant rs10069690, which was previously described as correlated with several cancer types by genome-wide association studies [18];
2. m*hTERT* is an epigenetic mechanism involved in both MCPyV (+) and (−) MCC, but had a different distribution when compared with specific clinical-pathological features (age, angioinvasion, Ki67, and PD-L1 expression);
3. m*hTERT*_high_ was related to a higher mortality risk when adjusted for the main clinico-pathological features.

hTERT is the functional subunit of the enzyme TERT and it is crucial to erase senescence and immortalize tumor cells [13–15]. Numerous epigenetic mechanisms are involved in *hTERT* expression, and among these, one of the most studied is its promoter methylation [13–15]. For the majority of the genes, hypo-methylation of the promoter is fundamental to permit an active transcription [13–15]. Nevertheless, several studies showed that hypermethylation of *hTERT* was paradoxically associated with higher *hTERT* mRNA levels and telomerase activity depending on the region of interest investigated [13–32]. Although several mechanisms cooperate in determining the final telomerase activity, promoter methylation proved to be one of the most relevant ones, singly affecting survival, recurrence, and progression in pediatric brain tumors, meningiomas, head and neck squamous cell carcinoma, leukemias, prostatic adenocarcinoma, and several other tumors [17–32]. In our series, we investigated not only the promoter but also a region of intron 4–5, where the SNP rs10069690 has been mapped (m*hTERT*) [18, 19]. This locus was previously associated with estrogen- and progesterone receptor-negative breast cancer, glioma, prostate, testicular germ cell, pancreas, and urinary bladder [18, 19]. It is becoming clear that regions with variable methylation tend to be mapped also in the gene body, overlapping known regulatory elements enriched for disease-associated SNPs [18, 19, 49]. These results are promising from a therapeutic point of view, for the availability of therapies inducing demethylation and repression of *hTERT* expression, as just shown in carcinoma cell lines and other in vitro studies with the demethylating agent [50, 51]. This is the first study that investigated frequency, association with clinical-pathological features, and prognostic relevance of m*hTERT* within the gene body (intron 4–5) in MCC patients. Xie et al. showed that *hTERT* expression is widespread in MCC and higher *hTERT* mRNA levels are associated with a shorter OS [9]. Furthermore, the only two genetic mechanisms analyzed by the authors were promoter mutation (1/6 cell lines and 4/35 MCC cases) and gene amplification (1/6 cell lines and 11/14 MCC cases) [9]. As clarified by the same authors, these two genetic alterations could only partially explain the diffuse *hTERT* mRNA expression (6/6 cell lines and 41/43) and telomerase activity (6/6 cell lines and 11/11 MCC cases) [9]. Unfortunately, for tissue overfixation of several enrolled cases, we were not able to obtain good quality RNA to monitor *hTERT* gene expression level, so we could not correlate it with DNA methylation. In line with these findings, other studies showed that *hTERT* promoter mutation is infrequent in MCC and suggested that other epigenetic mechanisms are probably involved [16, 17]. Our series confirmed these data as only three cases showed the mutation C250T (c.-146C>T) with very low allele frequency below 7%. The first main finding coming out from our study is that m*hTERT*_high_ is an epigenetic mechanism frequently involved in MCC (30/69, 43.5%). Although this result needs to be validated in larger case series, it suggests that m*hTERT* could be one important epigenetic mechanism involved in *hTERT* regulation, cooperating with other ones to justify the high levels of *hTERT* mRNA and telomerase activity found in these patients. The second main finding is the prognostic impact of m*hTERT*_high_, as just observed in other tumors [17–32]. In the multivariable model, we found that only m*hTERT*_high_ and p63 affected OS, with marginal significance for angioinvasion and absence of MCPyV. In addition, Kaplan-Meier analyses showed that m*hTERT*_high_ displays its effect on survival since about 12 months after the surgery. This finding suggests that m*hTERT*_high_ could be a middle- and long-term response predictor, as shown for other pathological features [8]. In addition, we found that m*hTERT*_high_ was a strong prognostic factor regardless its association with other unfavorable and favorable prognostic factors. Xie et al*.* found that, despite *hTERT* mRNA levels were associated with a shorter OS and they were strongly influenced by promoter mutation and gene amplification, these two genetic mechanisms did not affect the prognosis when singly evaluated [9]. This result is in line with what found by other authors in other tumors, where *hTERT* mRNA levels but not m*hTERT* promoter methylation affects the prognosis [30, 31]. A possible explanation, provided by the same authors and supported by us, is that mechanisms specifically regulating the levels of h*TERT* mRNA are differently involved in each tumor [9, 13–30]. This would explain why *hTERT* mRNA levels affect prognosis in almost all tumors but a singly evaluated mechanism could do it or not [9, 13–32]. Additionally, *TERT* promoter mutations have been shown to be spatially heterogeneous/sub-clonal in some tumors (follicular thyroid carcinoma, follicular thyroid tumors of uncertain malignant potential and meningiomas) and several genes (*MLH1*, *MGMT*, *CDKN2B*) could exhibit spatially heterogeneous/sub-clonal methylation patterns in different tumors (glioblastoma, breast carcinoma) [52–58]. Although these aspects have never been investigated in MCC, it is not possible to exclude that m*hTERT* and promoter methylation could be spatially heterogeneous/sub-clonal in this tumor and, as result, our data could be affected by the tissue/block selection. Future studies adopting single-cell sequencing will help to clarify if m*hTERT* and promoter methylation are heterogeneous/sub-clonal in MCC and if there are small fraction of tumor cells (i.e., tumoral stem cells) which could acquire *hTERT* demethylation in one allele causing small changes in overall DNA methylation. It is largely accepted that MCPyV and UV-radiations identify two divergent pathogenetic pathways in MCC, with many differences in the genetic landscape [5–8]. MCPyV (−) MCCs have a high mutation burden, frequent mutations in *RB1*, *TP53*, genes involved in chromatin remodeling (*ASXL1*, *MLL2*, and *MLL3*), and DNA damage [5–8]. By contrast, MCPyV (+) MCCs have a low mutation burden, reduced expression of H3K27me3, and no mutations in the previous listed genes [5–8]. Nevertheless, in both subgroups, there are involvement of common transcription factors (NFAT, P-CREB, and P-STAT3) and mutations in PI3K (*HRAS*, *KRAS*, *PIK3CA*, *PTEN*, and *TSC1*) and Notch pathways genes (*Notch1* and *Notch2*), supporting the presence of shared oncogenic pathways in both MCPyV (+) and (−) MCCs [5]. We did not find different distribution of m*hTERT* levels between MCPyV (+) and (−) cases, suggesting that m*hTERT* belong to the shared oncogenic pathways. This hypothesis is supported by the absence of correlation between m*hTERT* and site (Table 1), with this latter known to be associated with the pathogenetic pathway [trunk: MCPyV (+); head-and-neck and extremities: MCPyV (−)] [3]. According to our findings, Chteinberg E et al. found that DNA methylation age (DNAmAge) is not associated with MCPyV in MCC, as previously described for other viral infections as HPV or EBV [59]. In addition, Xie et al. found that absence of virus was associated with promoter mutation (in line with the higher mutation burden of MCPyV (−) MCC) but none with gene amplification and *hTERT* mRNA levels, supporting that *hTERT* could be involved in both pathogenic pathways [9].

## Conclusion

In conclusion, our study showed that m*hTERT* in intron 4–5 in proximity of the SNP rs10069690 strongly affects the prognosis of MCC patients, being involved in both MCPyV (+) and (−) tumors, cooperating with other epigenetic and genetic mechanisms as gene amplification in determining the final levels of *hTERT* mRNA and telomerase activity. Future studies with larger case series are needed to clarify the relationship between m*hTERT*, the other genetic and epigenetic mechanisms involved in the *hTERT* regulation, the pathogenesis (MCPyV and UV-radiations), and prognosis.

## Data Availability

The authors declare transparency and availability of data, material, and code (all data that support the findings of this research is deposited in a public repository).
